# Ultrastructural Study of Platelet Behavior and Interrelationship in Sprouting and Intussusceptive Angiogenesis during Arterial Intimal Thickening Formation

**DOI:** 10.3390/ijms222313001

**Published:** 2021-11-30

**Authors:** Lucio Díaz-Flores, Ricardo Gutiérrez, Maria Pino García, Miriam González-Gómez, Lucio Díaz-Flores, Sara Gayoso, Jose Luis Carrasco, Hugo Álvarez-Argüelles

**Affiliations:** 1 Department of Basic Medical Sciences, Faculty of Medicine, University of La Laguna, 38071 Tenerife, Spain; histologia54@gmail.com (R.G.); mirgon@ull.es (M.G.-G.); ldfvmri@yahoo.com (L.D.-F.J.); pilargon59@gmail.com (S.G.); jcarraju@ull.edu.es (J.L.C.); hargue@ull.es (H.Á.-A.); 2 Department of Pathology, Eurofins Megalab–Hospiten Hospitals, 38100 Tenerife, Spain; mpgarcias@megalab.es; 3 Instituto de Tecnologías Biomédicas de Canarias, University of La Laguna, 38071 Tenerife, Spain

**Keywords:** platelets, intimal thickening, peg-and-socket junctions, sprouting angiogenesis, intussusceptive angiogenesis, vascular regression, ultrastructure

## Abstract

Platelets in atherosclerosis, bypass stenosis, and restenosis have been extensively assessed. However, a sequential ultrastructural study of platelets in angiogenesis during the early phases of these lesions has received less attention. Our objective was the study of platelets in angiogenesis and vessel regression during intimal thickening (IT) formation, a precursor process of these occlusive vascular diseases. For this purpose, we used an experimental model of rat occluded arteries and procedures for ultrastructural observation. The results show (a) the absence of platelet adhesion in the de-endothelialized occluded arterial segment isolated from the circulation, (b) that intraarterial myriad platelets contributed from neovessels originated by sprouting angiogenesis from the periarterial microvasculature, (c) the association of platelets with blood components (fibrin, neutrophils, macrophages, and eosinophils) and non-polarized endothelial cells (ECs) forming aggregates (spheroids) in the arterial lumen, (d) the establishment of peg-and-socket junctions between platelets and polarized Ecs during intussusceptive angiogenesis originated from the EC aggregates, with the initial formation of IT, and (e) the aggregation of platelets in regressing neovessels (‘transitory paracrine organoid’) and IT increases. In conclusion, in sprouting and intussusceptive angiogenesis and vessel regression during IT formation, we contribute sequential ultrastructural findings on platelet behavior and relationships, which can be the basis for further studies using other procedures.

## 1. Introduction

Platelet participation in several pathophysiological mechanisms involved in atherosclerosis, bypass stenosis, and restenosis is an important subject of research in human and experimental pathology [1,2,3,4,5,6,7,8,9,10,11,12,13,14]. Platelets act in angiogenesis [15,16,17,18], which is one of the main pathophysiological mechanisms of this pathology. Platelet ultrastructure has been described in several conditions, including some of these processes [19,20,21,22,23]. However, there has been no sequential ultrastructural study on the behavior and relationship of platelets in the angiogenic and neovessel regressive/remodeling response during the early development of these occlusive vascular diseases, in which intimal thickening (IT) is an important lesional precursor and amplifier.

Arterial IT occurs in normal and pathological conditions and is made up of neointimal (myointimal) cells and newly formed extracellular matrix. It is located between the endothelium and the internal elastic lamina of the artery. In experimentally occluded arterial segments, we observed IT formation, in which angiogenic and neovessel regressive transitory phenomena participated (23). The main types of angiogenesis involved in IT development were sprouting and intussusceptive angiogenesis, which are the two principal and complementary forms of angiogenesis, with synergistic interaction. Neovascularization occurred in the first days of the onset of the injury and was rapidly followed (also in days) by regression of the newly formed vessels. The neovessels originated from the periarterial microvasculature by sprouting angiogenesis, and the intussusceptive angiogenesis was observed when the neovessels reached the arterial lumen. Experimental IT in occluded arterial segments, in which these events develop, can therefore be a useful substrate for the ultrastructural study of platelets during angiogenesis and vessel regression in tge early stages of the occlusive arterial pathology.

Given the above, our objective was the sequential ultrastructural study of the behavior and relationship of platelets in pre- and early sprouting, and intussusceptive angiogenesis and neovessel regression during IT formation. To that end, we used an experimental model (occluded arteries) that reproduced most of the findings that occur in human pathology during the formation and remodeling of this process.

## 2. Results

### 2.1. Controls

**Figure 1:**
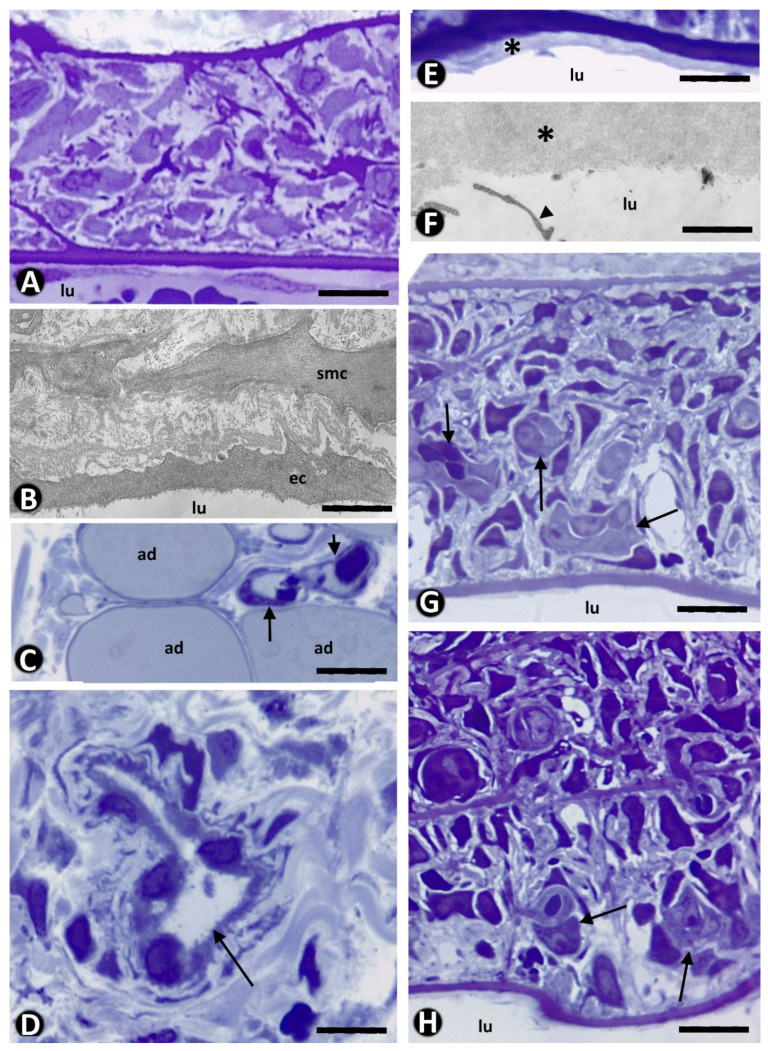
(**A**–**D**). Control: Image of an unmodified artery is observed in semithin and ultrathin sections (**A**,**B**). Normal microvasculature in the connective and adipose periarterial tissues is also seen (**C**,**D**, arrows). A normal vessel and a few interstitial mononuclear cells are present in the periarterial tissues at the end of a sham-operated arterial segment (**D**). (**E**–**H**): Initial stage in occluded arteries. De-endothelialized arteries are observed without platelet adhesion. Note the extracellular matrix (asterisk) and absence of adhered platelets or re-endothelialization (**E**,**F**). Occasional cell debris are present (**F**, arrowhead). Absence of platelet adhesion is observed even when neovessels are present in the artery wall (arrows) without crossing the internal elastic membrane of the artery (**G**,**H**). Arterial lumen: lu. Adipocytes: ad. Endothelial cell: ec. Smooth muscle cell: smc. (**A**,**C**,**D**) and (G,H): Semithin section. Toluidine blue staining. (**B**,**F**): Ultrathin section: Uranyl acetate and lead citrate. Bar: (**A**,**G**,**H**): 15µm. (**B**): 6µm. (**C**,**D**): 20 µm. (**E**): 10 µm. (**F**): 2 µm.

The structure of the arteries remained unmodified (Figure 1A,B) and capillaries were not observed in the arterial wall (Figure 1A). Platelet-related changes were not seen in the lumen of the artery or in the arterial wall. In the connective and adipose tissues surrounding the artery, the microvasculature was also within normal limits (Figure 1C,D), and only a few neutrophils and mononuclear cells were present around the ends of the sham-operated arterial segments (Figure 1D).

### 2.2. Platelets in Angiogenesis and Neovessel Regression during Arterial IT Formation

For better exposure of platelet characteristics and relationships, we have divided the description of the results into two phases, initial and secondary, taking into account the evolution over time of the occluded arterial segments. However, some of the facts may overlap in both phases.

#### 2.2.1. Initial Phase. Platelets during Sprouting and Intussusceptive Angiogenesis

In the initial phase (days 1–9), we observed (a) de-endothelialization and slight cellular damage in the vessel media and adventitia, with the absence of initial platelet adhesion to the de-endothelialized surface and re-endothelialization (days 1–3) and (b) the presence of platelets in the lumen of neovessels during sprouting angiogenesis toward the artery wall (days 4–6) and intussusceptive angiogenesis in the arterial lumen with initial IT formation (days 6–9).

Although de-endothelialization was observed, the initial phenomena of platelet adhesion to the de-endothelialized surface and re-endothelialization did not occur in these occluded arterial segments (Figure 1E,F), which were isolated from the circulation. The absence of platelet adhesion and re-endothelialization remained even when neovessels were present in the media layer without penetrating the internal elastic membrane (Figure 1G,H).

**Figure 2:**
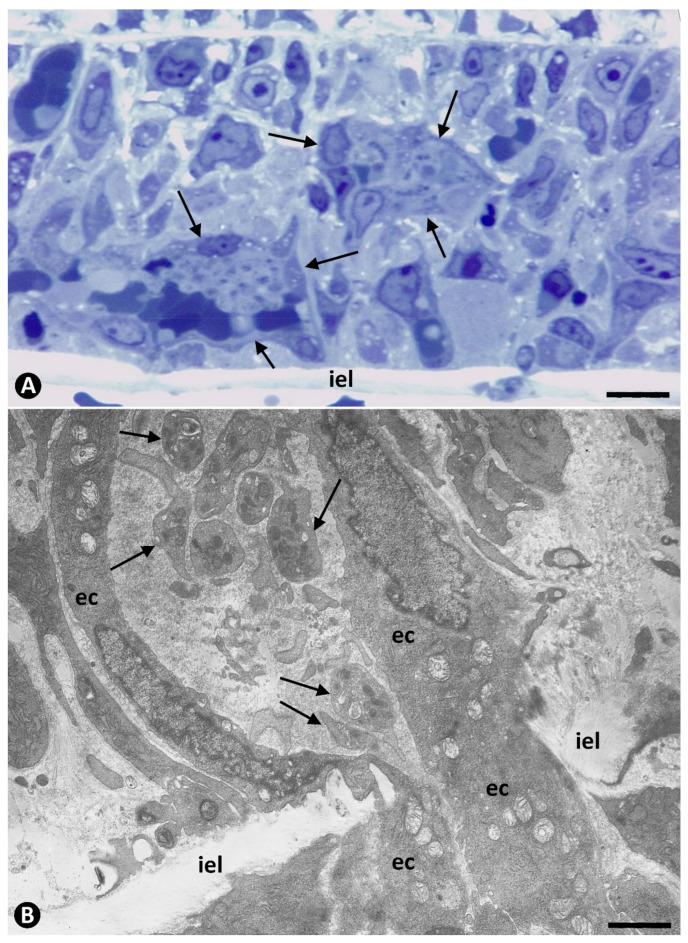
Platelets in newly formed vessels that cross the wall of occluded arteries. (**A**): Semithin section in which numerous platelets, independent of each other, are observed in neovessels (arrows) penetrating the media layer and retained by the internal elastic membrane (iel) of an artery. (**B**): Ultrastructural image in which a vessel with intraluminal platelets (arrows) crosses the internal elastic lamina (iel), acquiring an hourglass appearance. Endothelial cell: ec. (**A**): Semithin section. Toluidine blue staining. (**B**): Ultrathin section: Uranyl acetate and lead citrate: Bar: (**A**): 15 µm (**B**): 3.5 µm.

Large numbers of platelets were observed in neovessels penetrating the wall of the occluded arteries from the periarterial microvasculature (sprouting angiogenesis from vasa-vasorum). Whether or not they were associated with red blood cells, the platelets were independent of each other and frequently occupied the entire lumen of some neovessels, which were dilated (Figure 2). Many of these penetrating vessels containing platelets and red blood cells are retained by the internal elastic membrane of the arteries (Figure 2A) or appear passing through its fenestrations (Figure 2B). When the neovessels pass through the fenestrations of the internal elastic membrane, they take on an hourglass appearance (Figure 2B) and contribute numerous platelets, red blood cells, ECs, and periendothelial cells, as well as some leukocytes and abundant fibrinous material, to the isolated arterial lumen.

**Figure 3:**
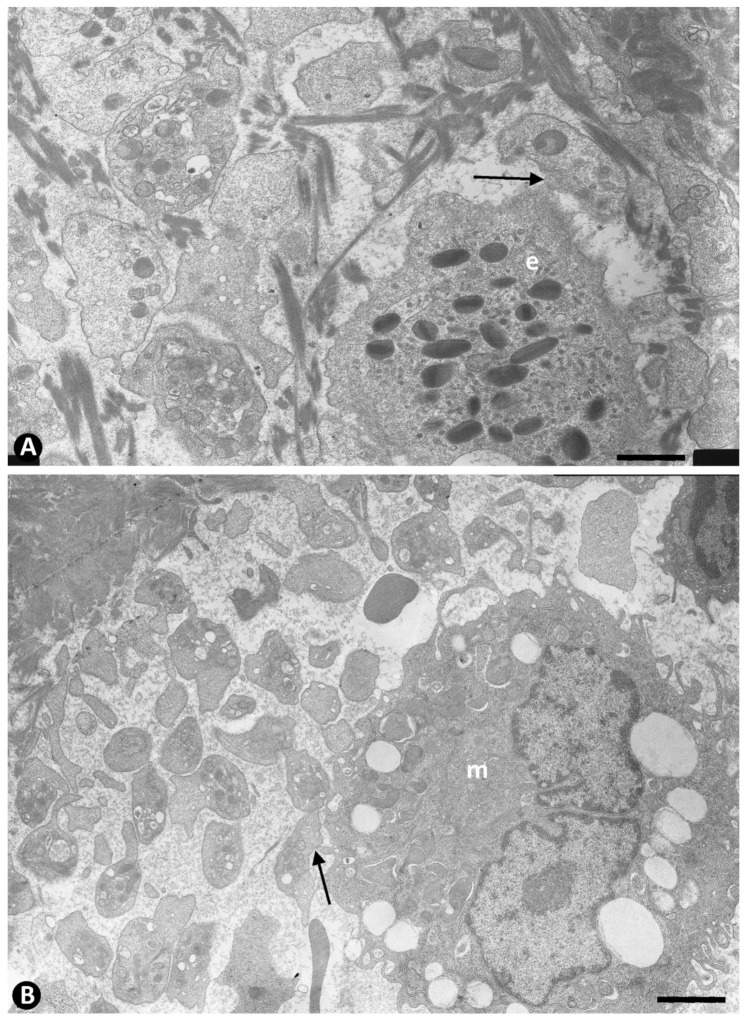
Association of platelets with blood components contributed to the lumen of occluded arteries from the periarterial microvasculature. (**A**): Relationship of the platelets with fibrinous material and one eosinophil (e). Note the characteristic granules in the latter. (**B**): Relationship of platelets with a macrophage (m). Arrows point to contacts between platelets and the eosinophil and the macrophage. Ultrathin sections. Uranyl acetate and lead citrate. Bar: (**A**): 1 µm, (**B**): 3.5 µm.

**Figure 4:**
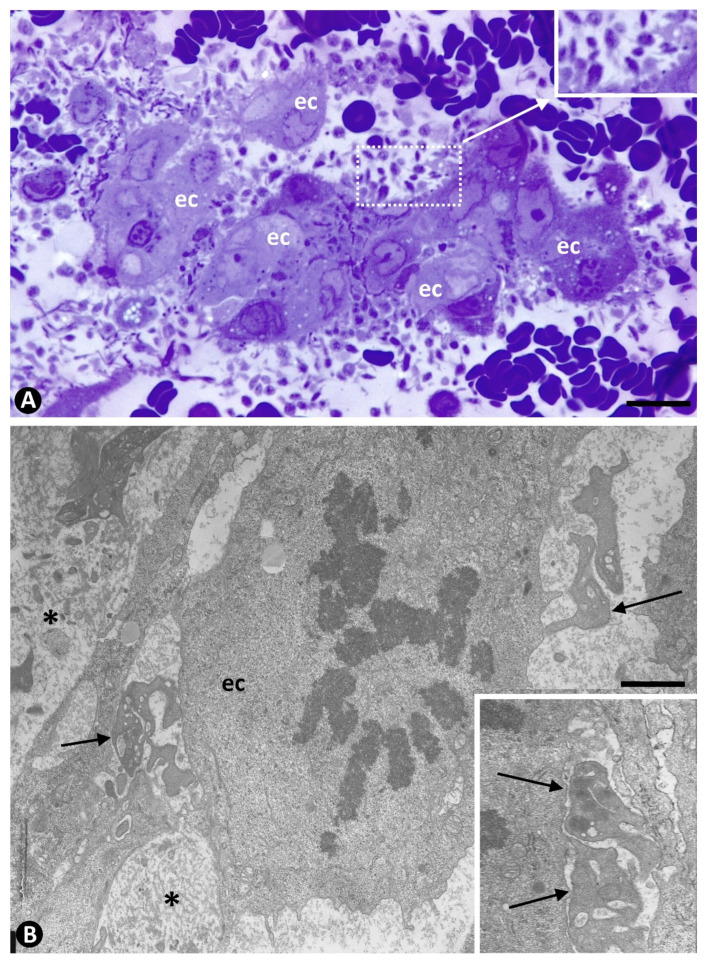
Association of platelets with ECs contributed to the lumen of occluded arteries from the periarterial microvasculature. (**A**): Semithin section in which platelets are observed in association with a portion of an EC aggregate. Note that platelets are present around the aggregate and between the ECs (ec). Insert in (**A**): A group of platelets at higher magnification. (**B**): Platelets are seen in the abluminal and luminal surfaces of the ECs that begin their polarization in EC aggregates. This image corresponds to an EC in mitosis (ec). In an abluminal space around ECs presence of membranous material (asterisks). Insert of (**B**), a detail of the relationship of platelets and another mitotic EC. (**A**): Semithin section. Toluidine blue staining. (**B**): Ultrathin section. Uranyl acetate and lead citrate. Bar: (**A**): 15 µm, (**B**): 3.5 µm.

**Figure 5:**
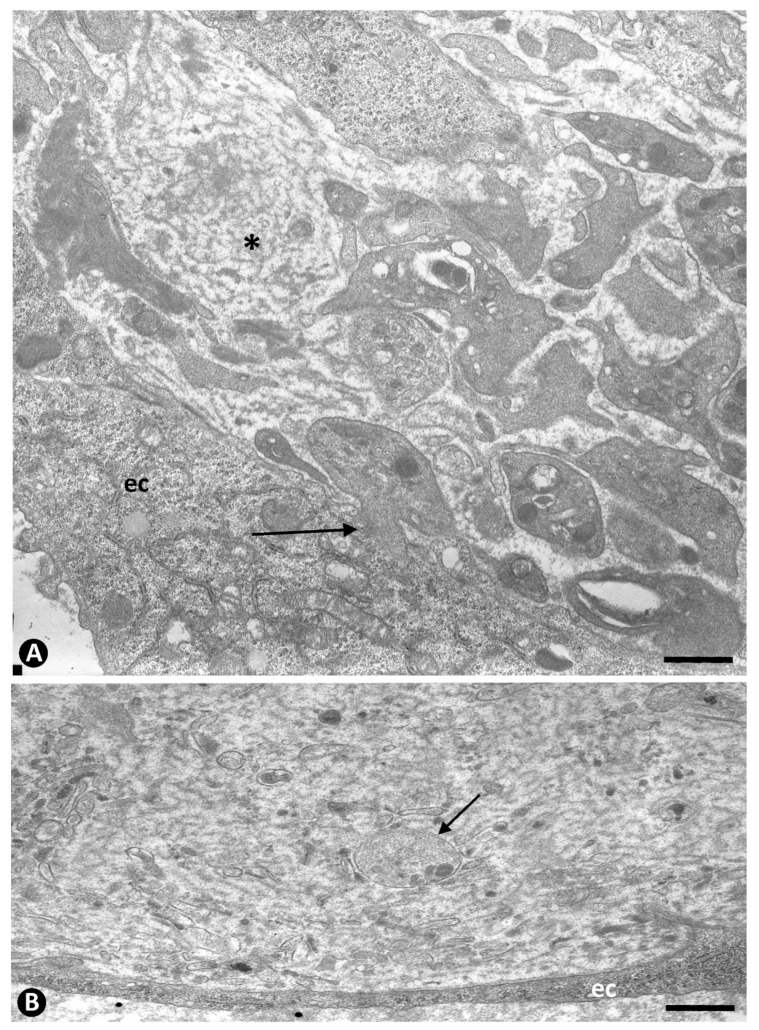
Platelets in the abluminal space around ECs. (**A**): Platelets with a different morphology are observed in association with newly formed basal membrane-like material arranged in multiple layers resembling fingerprints (asterisk). Note a platelet with a projection penetrating an EC (arrow) (compare with peg-and-socket junctions in Figure 6). (**B**): Presence of granules, similar to those in a modified platelet (arrow), is observed between the membrane-like material in the abluminal space around the EC. Ultrathin sections. Uranyl acetate and lead citrate. Bar: (**A**,**B**): 1 µm.

**Figure 6:**
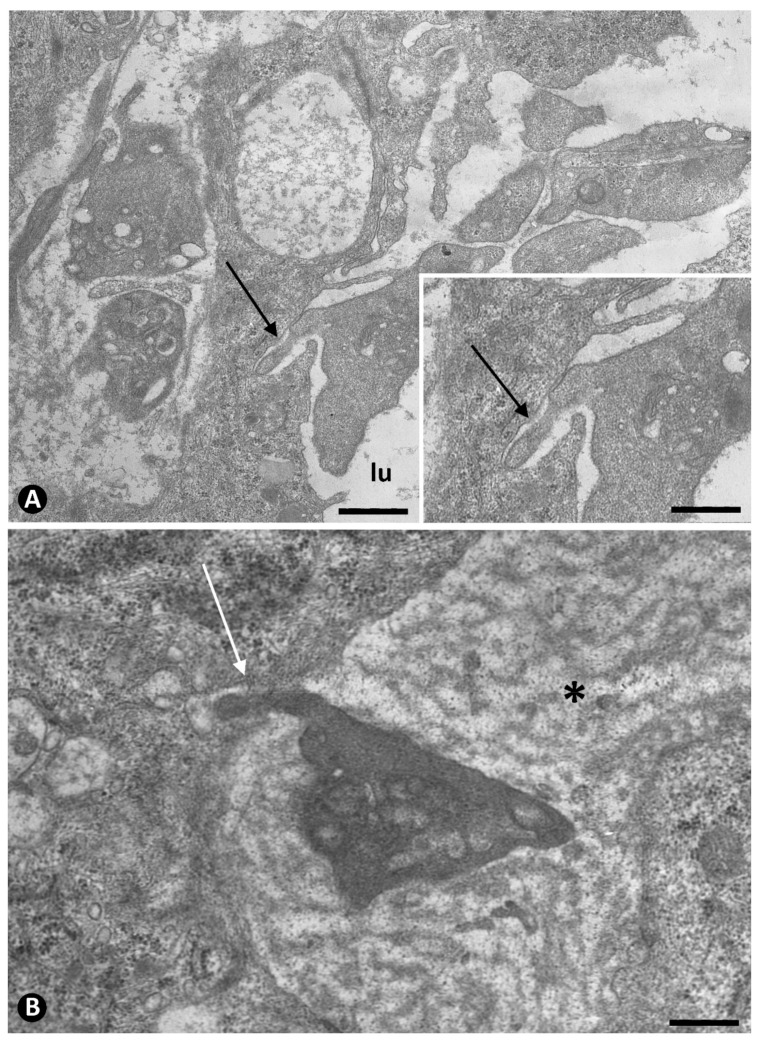
Presence of peg-and-socket junctions between platelets and ECs in the luminal and abluminal EC surfaces. (**A**): A platelet pseudopod forming a peg is observed penetrating an invagination of the luminal surface of an EC (the socket) (arrow). Insert: A detail at higher magnification of the peg and socket (arrow). (**B**): A similar peg and socket between a platelet and an EC, but in the abluminal surface of the EC (arrow). Note the fingerprint-like membranous material (asterisk)l around the platelet. The membranous material with or without blood components (e.g., platelets) forms the core, and the surrounding ECs the cover, of the intussusceptive pillars in the arterial lumen (**A**,**B**). Ultrathin sections. Uranyl acetate and lead citrate. Bar: (**A**): 0.7 µm, (**B**): 0.5 µm.

Prior to the initiation of intussusceptive angiogenesis, numerous platelets in the lumen of occluded arteries were associated with the other components contributed by the neovessels that cross the internal elastic membrane, including fibrinous material (Figure 3A), eosinophils (Figure 3A), neutrophils, macrophages (Figure 3B), and aggregates of ECs (EC spheroids) (Figure 4). The platelets in the EC aggregates were seen around them and intermixed with their ECs, which were non-polarized (Figure 4). As the ECs in the aggregates reorganized with luminal and abluminal polarization, platelets were present in the corresponding neoformed luminal and abluminal spaces, even in ECs in mitosis (Figure 4B). In the abluminal spaces, platelets intermingled with a newly formed basal membrane-like material arranged in multiple layers, resembling fingerprints (Figure 5A). Isolated granules, suggesting platelet provenance, were also observed between this abluminal material (Figure 5B). The platelets and interstitial membranous material formed the core, and the surrounding ECs formed the cover of developing pillars (hallmark of intussusceptive angiogenesis) in the lumen of the arteries (Figure 6).

Contacts between platelets and ECs were observed in both luminal and abluminal surfaces of the ECs. Importantly, finger-shaped protrusions from platelets were seen penetrating invaginations in the luminal and abluminal surfaces of ECs, originating a peg-and-socket-type junction, in which the platelet formed the peg, and the EC formed the socket (Figure 6A,B). Thus, the platelet peg was mainly formed through a pseudopod invaginating into an EC, while the EC formed the socket through a suitable invagination for the size of the peg. The platelet pegs showed varying lengths of penetration; no organelles were observed (Figure 6A,B).

#### 2.2.2. Secondary Phase. Platelets during Regression of the Newly Formed Vessels

**Figure 7:**
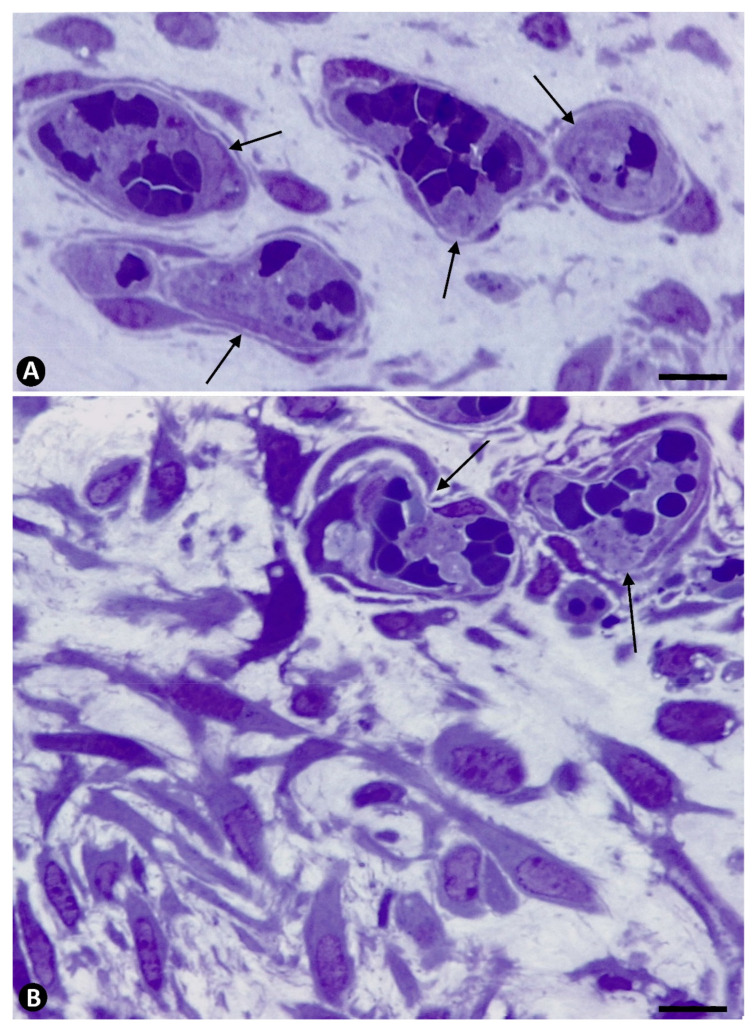
Platelets during regression of the newly formed vessels. (**A**): Aggregates of platelets and red blood cells in regressive vessels (arrows). (**B**): Numerous myointimal cells proliferating around regressive vessels with aggregates of platelets and red blood cells (arrows). Semithin sections. Toluidine blue staining. Bar: (**A**,**B**): 15 µm.

**Figure 8:**
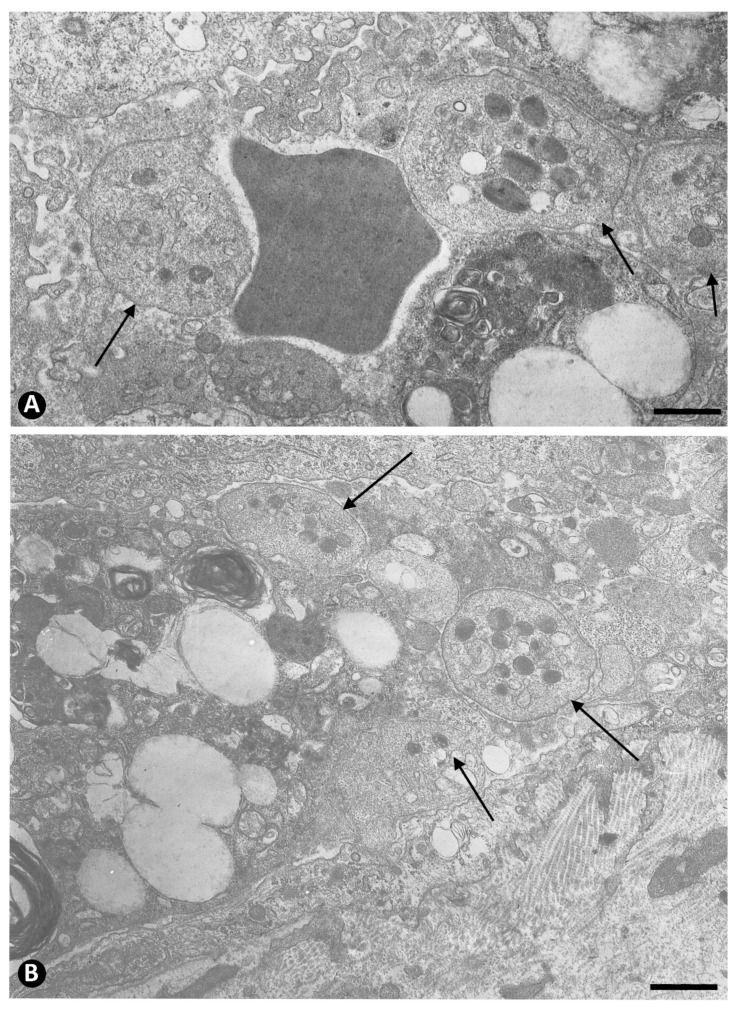
Ultrastructural images of aggregates of platelets (arrows) in regressive vessels. Degenerative phenomena are observed in surrounding cells. Ultrathin sections. Uranyl acetate and lead citrate. Bar: (**A**,**B**): 0.7 µm.

**Figure 9:**
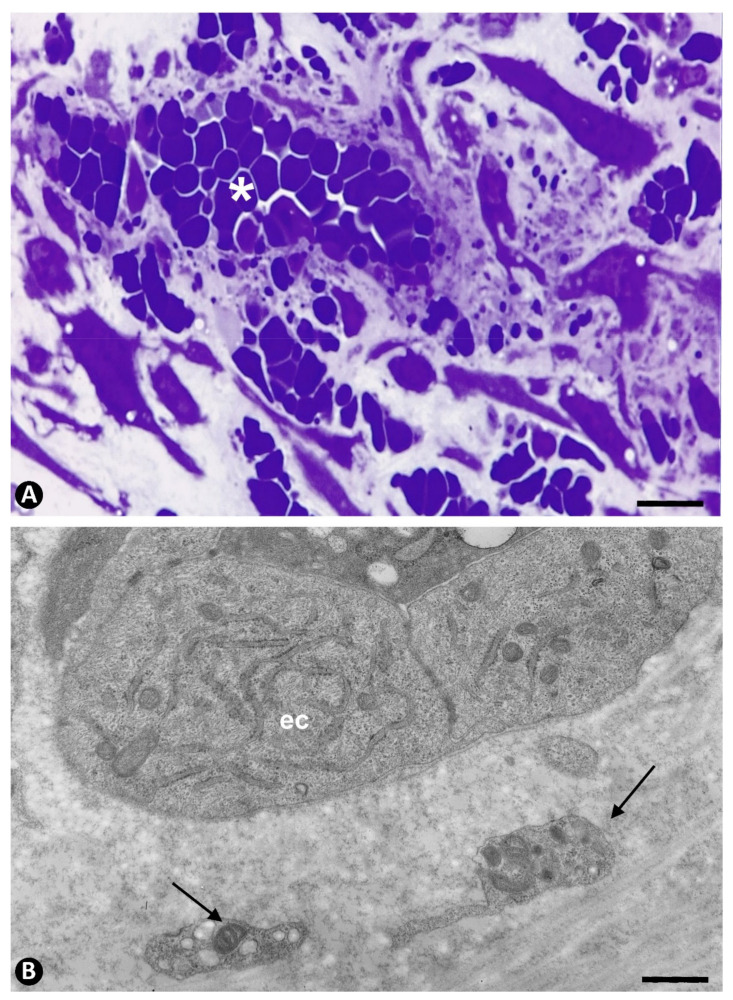
Numerous extravasated platelets around a regressive vessel plugged by an intraluminal accumulation of red blood cells (asterisk). Note proliferated myointimal cells in the interstitium. (**B**): Ultrastructural image of extravasated platelets (arrows) around a regressive vessel. Endothelial cell: ec. (**A**): Semithin section. Toluidine blue staining. (**B**): Ultrathin section. Uranyl acetate and lead citrate. Bar: (**A**): 15 µm, (**B**): 1 µm.

In the secondary phase (days 10–18), platelet aggregation in the lumen of the newly formed vessels occurred, with regression of most of these neovessels and increased IT. Thus, the platelets in these regressive vessels were agglutinated and frequently associated with varying numbers of red blood cells (Figure 7A,B and Figure 8A,B). Degenerative phenomena were seen in the regressive vessels (Figure 8A,B). This platelet aggregation coincided with the presence of abundant perivascular (myointimal, intimal) cells in the core of pillars and in the interstitium. Immediately, the development of one or several persistent preferential vessels occurred, generally in the axis of the arterial segment, which was followed by the proliferation of myointimal cells and extracellular matrix formation, leading to increased IT. Platelets extravasated from regressive neovessels were also observed in the interstitium (Figure 9).

## 3. Discussion

**Figure 10:**
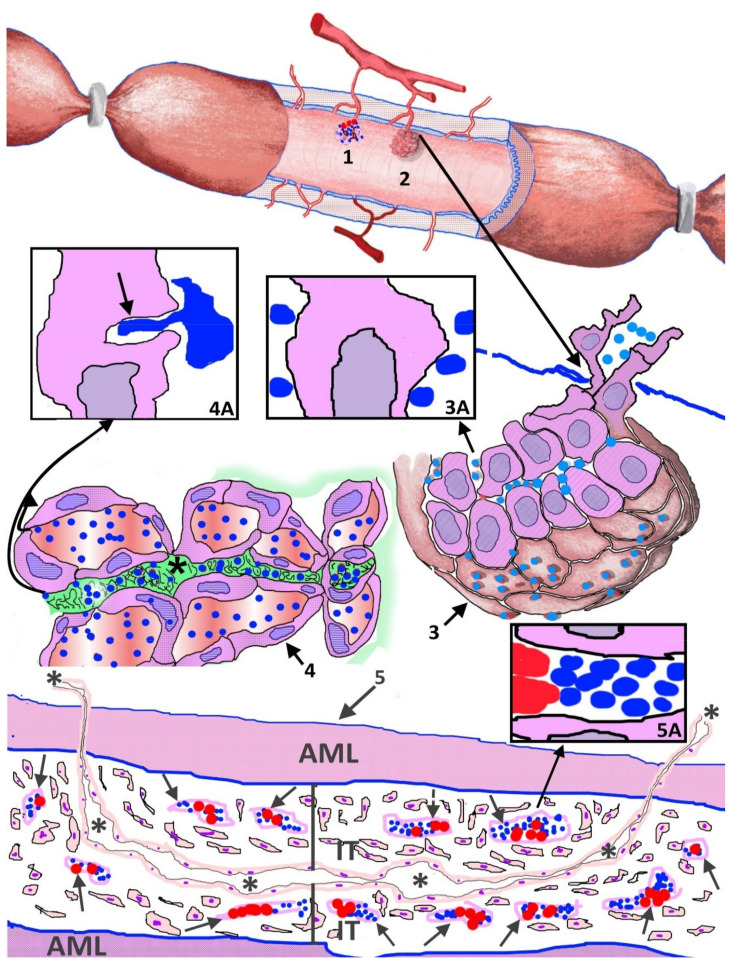
Schematic representation of platelet behavior and interrelationship in occluded arteries. (1) Contribution of platelets and other blood components (with which platelets establish interrelations) to the lumen of the occluded arterial segment, from newly formed vessels, which cross the arterial wall from the periarterial microvasculature, in which they originate by sprouting angiogenesis. (2,3,3A) Incorporation of platelets (colored in blue) between non-polarized ECs that form aggregates (EC spheroids) in the lumen of the artery. (4, 4A) Reorganization of EC aggregates with (a) persistence of platelets in the luminal and abluminal spaces when the ECs are polarized, establishing peg-and-socket junctions with ECs in both spaces (4A, arrow) and (b) platelet participation in the formation of intussusceptive pillar cores together with basal-membrane-like material in the abluminal space (asterisk). (5, 5A) Aggregation of platelets in the lumen of regressive neovessels (‘paracrine transitional organoid’) (arrows) coinciding with myointimal cell proliferation and intimal thickening increase. Formation of a preferential vessel (asterisks). AML: Arterial media layer.

In this work, we report the ultrastructural behavior and relationship of platelets in experimentally occluded arteries, in which important pathophysiological mechanisms occur, including sprouting and intussusceptive angiogenesis and IT formation. The findings can be summarized as follows (Figure 10): (a) the absence of both platelet adhesion and re-endothelialization in the luminal surface of the artery after de-endothelialization, (b) the presence of numerous platelets in the lumen of newly formed vessels, which arise by sprouting angiogenesis from the periarterial microvasculature and penetrate the arterial wall, (c) the contribution to the artery lumen of myriad platelets, as well as ECs, perivascular cells, and other blood components, from the newly formed vessels after crossing the arterial wall, (d) the interrelationship of platelets with these components, (e) the incorporation of platelets around intraarterial, newly formed EC aggregates (EC spheroids) and between their non-polarized ECs, (f) the existence of platelets in the luminal and abluminal spaces around ECs when they acquire polarization, (g) interconnections between platelets and ECs, with the establishment of a special type of union of platelets with ECs (peg-and-socket junctions) in both spaces, (h) the persistence of platelets in the abluminal space, contributing, together with basal membrane-like material and granules, to form the core of initial intussusceptive pillars, in which the cover corresponds to the surrounding ECs, and (i) the aggregation of platelets in the lumen of regressive neovessels, coinciding with myointimal/intimal cell proliferation and IT formation.

Some of the previously mentioned platelet findings are remarkable. Platelet adhesion to extracellular matrix at sites of vascular injury and platelet activation have been extensively studied and demonstrated [24,25,26,27,28]. However, after de-endothelialization, probably due to ischemia [29,30], the absence of platelet adhesion and re-endothelialization can be logically due to the impossibility of platelet and EC contribution from interrupted circulation and ECs from the ends of the occluded arterial segment. Therefore, the contribution of blood components, including numerous platelets, immune cells, and ECs, to the arterial lumen only occurs from newly formed vessels from the periarterial microvasculature that cross the arterial wall. Intimal cells can be originated from pericytes of these penetrating vessels and from smooth muscle cells of the media layer of the artery.

The interrelationship of platelets and immune cells and fibrin concurs with descriptions by other authors in several conditions [31,32,33,34,35,36]. Although the interrelationship between platelets and ECs has also been well studied [37,38,39], this interrelationship requires particular attention when platelets are incorporated and intermixed in the arterial lumen with ECs, since platelet and ECs establish peg-and-socket junctions. Recently, we observed structures that suggest the existence of this type of junction between platelets and ECs [23]. We now confirm its existence and characteristics in a broader and more specific study. The presence of this junction between platelets and ECs has not been described by other authors. Thus, when ECs penetrate the lumen of the artery, they lose their luminal and abluminal polarization and form multicellular aggregates, resembling the EC spheroids used in three-dimensional in vitro angiogenesis research and regenerative medicine [40,41,42]. Platelets are present in these aggregates, and when the ECs become polarized again, with the presence of luminal and abluminal spaces, platelets persist in both locations. The platelets and ECs in these locations establish peg-and-socket junctions, in which the pseudopodes of platelets form the peg, and EC surface invaginations form the socket. These junctions are similar to those described in certain conditions between pericytes or vascular smooth muscle cells and ECs (pericytes or vascular smooth muscle cells also form the peg and ECs form the socket) [23,43,44,45,46].

The granules observed between the basal membrane-like material in the abluminal space around the ECs are difficult to interpret ultrastructurally. They are similar to some granules in involutive platelets located in the vicinity. Activated platelets release cargo from their storage (granule exocytosis) [47,48,49,50]. In this case, the identification of whole granules in the interstitium requires future studies.

We demonstrated pillar formation in the lumen of the occluded arteries from the EC spheroids in previous work [23]. These pillars met the conditions for consideration as intussusceptive pillars (pillars as the hallmark of intussusceptive angiogenesis) [51,52,53,54,55,56,57,58], including their appearance and disappearance in serial semithin sections [58]. The persistence of platelets in the core of early pillars can contribute to the incorporation of intimal cells by the release of platelet factors. Platelet interrelationship with ECs and with other cells and interstitial components during intussusceptive angiogenesis requires further studies with other procedures.

The presence of aggregates of platelets in the numerous newly formed vessels during their regression and their coincidence with a marked increase in IT suggest that the intravascular accumulations of these factor-releasing platelets act as a ‘paracrine transitional organoid’. Likewise, the presence of extravasated platelets in the interstitium from the regressive vessels may be due to the alteration of the endothelium of these regressive vessels, platelet migration, or both. Indeed, the migratory capacity of platelets has been described in some conditions [59,60,61].

In conclusion, we studied the sequential ultrastructural behavior and interrelationship of platelets in occluded arteries in sprouting and intussusceptive angiogenesis and neovessel regression during IT formation. We contribute to the existing literature with our findings of platelet (a) absence in early de-endothelialized arteries, (b) presence in the lumen of the neovessels penetrating the arterial wall from the periarterial microvasculature, (c) incorporation in the arterial lumen, (d) interrelationship with blood and interstitial components, especially with ECs (peg-and-socket junctions), (e) participation in pillar formation during intussusceptive angiogenesis, facilitating IT formation, and (f) arrangement in aggregates in the lumen of regressive neovessels, forming a ‘paracrine transitional organoid’ that facilitates interstitial cell proliferation with increased IT. Some of these observations can be the basis for further studies using other procedures.

## 4. Material and Methods

Adult Sprague–Dawley rats with an average weight of 300 g were used in accordance with the guidelines of the Ethics Committee of La Laguna University (La Laguna, Comité de Ética de la Investigación y de Bienestar Animal, CEIBA 2021-3077; 8 October 2021). The rats were fed standard rat chow and water unrestrictedly and maintained in pathogen-free conditions. During surgical procedures and tissue removal, the rats were anesthetized with ketamine (150 mg/Kg. i.p.). A surgical microscope was used to expose 1.5 cm segments of femoral arteries. In the rats (n: 40), ligatures with a 10/0 thread were applied to both the proximal and distal ends of the dissected femoral arterial segments, without damaging the surrounding microvasculature. A collateral present in these occluded femoral segments was also ligated 0.3 cm from the ostium. For control, rats (n:18) were sham-operated according to the microsurgical procedures described above, except for the application of ligatures.

Specimens from femoral arteries and surrounding connective tissue were removed daily from day 1 to 18. Specimens for electron microscopy (ultrathin sections) and semithin sections were initially fixed in glutaraldehyde solution, diluted to 2% with sodium cacodylate buffer, pH 7.4, for 6 h, at 4 °C. They were then washed in the same buffer, post-fixed for 2 h in 1% osmium tetroxide, dehydrated in a graded ethanol series, and embedded in epoxy resin. Serial semithin sections (1.5 μm) were mounted on acid-cleaned slides, stained with 1% Toluidine blue (Merck^®^, Darmstadt, Germany), and observed under a Leica^®^, Wetzlar, Germany, DM-750 light microscope, with an integrated High-Definition Camera (Leica^®^, Wetzlar, Germany, ICC 50W). Ultrathin sections were double-stained with uranyl acetate and lead citrate. The grids were examined at 60 kV with JEOL^®^ 100B and JEM 1011 Akishima, Tokyo, Japan, electron microscopes. The material (blocks/pyramids) of part of samples was obtained from our files throughout our studies in this field (since 1985) [62] and supplemented to obtain all the stages of this study. All the surgical procedures were undertaken by ourselves, and the blocks/pyramids were processed again for the current work. The material of the sham-operated rats/controls of our files (obtained in 1998) [63] were supplemented in the present work with new samples (days 13–18).

## Data Availability

All the data are reported in the present paper.
